# Glycemic Control, Renal Progression, and Use of Telemedicine Phone Consultations Among Japanese Patients With Type 2 Diabetes Mellitus During the COVID-19 Pandemic: Retrospective Cohort Study

**DOI:** 10.2196/42607

**Published:** 2023-11-21

**Authors:** Akiko Sankoda, Yugo Nagae, Kayo Waki, Wei Thing Sze, Koji Oba, Makiko Mieno, Masaomi Nangaku, Toshimasa Yamauchi, Kazuhiko Ohe

**Affiliations:** 1 Department of Planning, Information and Management The University of Tokyo Hospital Tokyo Japan; 2 Department of Diabetes and Metabolic Diseases Graduate School of Medicine The University of Tokyo Tokyo Japan; 3 Department of Biomedical Informatics Graduate School of Medicine The University of Tokyo Tokyo Japan; 4 Department of Biostatistics, School of Public Health Graduate School of Medicine The University of Tokyo Tokyo Japan; 5 Department of Medical Informatics Center for Information Jichi Medical University Shimotsuke Japan; 6 Division of Nephrology and Endocrinology Graduate School of Medicine The University of Tokyo Tokyo Japan

**Keywords:** glycemic control, renal progression, telemedicine, phone consultations, COVID-19, diabetes mellitus, type 2 diabetes

## Abstract

**Background:**

Reduced or delayed medical follow-ups have been reported during the COVID-19 pandemic, which may lead to worsening clinical outcomes for patients with diabetes. The Japanese government granted special permission for medical institutions to use telephone consultations and other remote communication modes during the COVID-19 pandemic.

**Objective:**

We aimed to evaluate changes in the frequency of outpatient consultations, glycemic control, and renal function among patients with type 2 diabetes before and during the COVID-19 pandemic.

**Methods:**

This is a retrospective single-cohort study conducted in Tokyo, Japan, analyzing results for 3035 patients who visited the hospital regularly. We compared the frequency of outpatient consultations attended (both in person and via telemedicine phone consultation), glycated hemoglobin A_1c_ (HbA_1c_), and estimated glomerular filtration rate (eGFR) among patients with type 2 diabetes mellitus during the 6 months from April 2020 to September 2020 (ie, during the COVID-19 pandemic) with those during the same period of the previous year, 2019, using Wilcoxon signed rank tests. We conducted a multivariate logistic regression analysis to identify factors related to the changes in glycemic control and eGFR. We also compared the changes in HbA_1c_ and eGFR from 2019 to 2020 among telemedicine users and telemedicine nonusers using difference-in-differences design.

**Results:**

The overall median number of outpatient consultations attended decreased significantly from 3 (IQR 2-3) in 2019 to 2 (IQR 2-3) in 2020 (*P*<.001). Median HbA_1c_ levels deteriorated, though not to a clinically significant degree (6.90%, IQR 6.47%-7.39% vs 6.95%, IQR 6.47%-7.40%; *P*<.001). The decline in median eGFR was greater during the year 2019-2020 compared to the year 2018-2019 (–0.9 vs –0.5 mL/min/1.73 m2; *P*=.01). Changes in HbA_1c_ and eGFR did not differ between patients who used telemedicine phone consultations and those who did not. Age and HbA_1c_ level before the pandemic were positive predictors of worsening glycemic control during the COVID-19 pandemic, whereas the number of outpatient consultations attended was identified as a negative predictor of worsening glycemic control during the pandemic.

**Conclusions:**

The COVID-19 pandemic resulted in reduced attendance of outpatient consultations among patients with type 2 diabetes, and these patients also experienced deterioration in kidney function. Difference in consultation modality (in person or by phone) did not affect glycemic control and renal progression of the patients.

## Introduction

In April 2020, the Japanese government declared a state of emergency in response to COVID-19, affecting the nation’s habits and lifestyle. This declaration resulted in various impacts, including social distancing and restrictions on daily movement, such as going out [1]. Diabetes mellitus (DM) is a known risk factor of severe COVID-19, and patients with DM have been encouraged to take precautions [2,3]. It was reported that drastic lifestyle changes during COVID-19 worsened glycemic control [4], and the overwhelming of health care systems caused a deterioration of chronic medical conditions [5]. Reports showed reduced or delayed hospital visits, with fear of catching the infection preventing patients from continuing in-person hospital visits [6-9]. Management of DM during the pandemic was critically important because patients with diabetes were reported to have higher probabilities of hospital admissions and deaths due to COVID-19 infection, compared to those without diabetes [10]. Evidence also showed that patients with DM were observed to experience progression of chronic kidney disease over a short period of time, warranting close monitoring of kidney function among these patients [11].

Telemedicine has expanded in many countries during the pandemic [12-14] to maintain access to health care services. The University of Tokyo Hospital started telemedicine consultation for the first time, using voice-only phone consultations, after Japan’s Ministry of Health, Labour, and Welfare granted special permission for medical care via telephone calls and other remote communication modes during the COVID-19 pandemic. With telemedicine consultations, physicians reviewed patients’ health conditions through phone interviews, provided lifestyle advice, and prescribed patients’ usual medicines for refill when health status was stable. When the physicians determined a need for further examinations, the patients were asked to visit the hospital for blood tests and physical examinations.

Before the pandemic, Japan’s government adopted a conservative strategy toward telemedicine, and the use of telemedicine for medical consultation has been limited [15]. Miyawaki et al [16] performed a telemedicine use survey among Japanese working-age population during COVID-19 and discovered a lower use rate of telemedicine, which was 4.7%. It was unknown if this newly introduced telemedicine model was well implemented among patients with diabetes, who were predominantly older patients. As continuity of care is imperative among patients with diabetes, there is a need to examine the utility of telemedicine among these patients as well as its impact toward disease control, such as glycemic control and renal function [17].

The primary objective of this study was to evaluate changes in the frequency of outpatient consultations, glycemic control, and renal function among a study cohort of patients with type 2 DM before and during the early phase of the COVID-19 pandemic (ie, April to September 2020). We also aimed to investigate the utilization rate of telemedicine via phone consultation. Next, we compared the glycemic control and renal function among telemedicine users and telemedicine nonusers during COVID-19.

## Methods

### Study Design

This is a single-center retrospective cohort study conducted at The University of Tokyo Hospital in Tokyo, Japan. The evaluation periods were from April to September 2019 and from April to September 2020.

### Study Population

**Figure 1:**
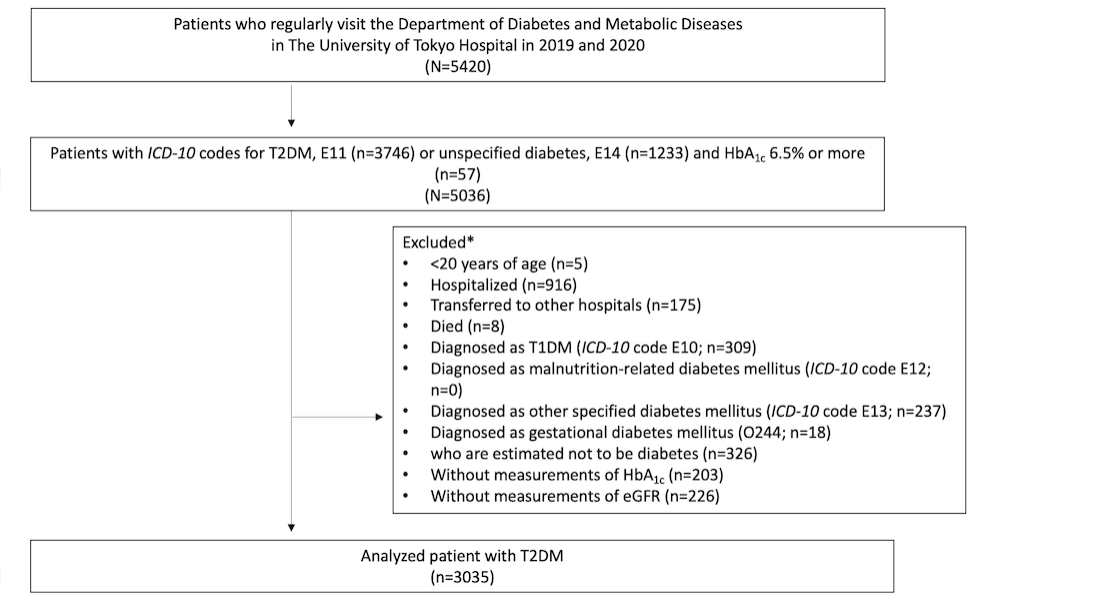
Recruitment of study population. eGFR: estimated glomerular filtration rate; HbA_1c_: hemoglobin A_1c_; *ICD-10: International Classification of Diseases, Tenth Revision*; T1DM: type 1 diabetes mellitus; T2DM: type 2 diabetes mellitus. *The excluded categories may have overlaps, as one patient could potentially fall into multiple categories.

Before the pandemic, patients usually visited the hospital every 1 to 3 months to check their hemoglobin A_1c_ (HbA_1c_), blood glucose, and similar metrics. During the pandemic, most patients continued with in-person hospital visits, though some chose telemedicine phone consultation in addition to in-person hospital visits. As the focus of our study was the impact of telemedicine use on disease management in adults, we excluded patients who were aged <20 years, transferred to other hospitals, had incomplete records, or experienced outcomes beyond routine disease management (eg, hospitalization, death, and change in diagnosis; Figure 1). We defined telemedicine users as patients who attended a telemedicine phone (voice) consultation with physicians at least once during the pandemic and telemedicine nonusers as patients who did not attend a telemedicine phone (voice) consultation with physicians at all.

### Data Collection Procedures

Demographic, clinical, and laboratory data were extracted from electronic health records. We extracted complications using the *International Classification of Diseases, Tenth Revision* (*ICD-10*) codes registered in the electronic health records, including dyslipidemia, hypertension, cardiovascular disease, chronic kidney disease, cognitive impairment, and malignancy (Table S1 in Multimedia Appendix 1). We collected age, sex, and medical comorbidities as the participants’ baseline characteristics.

### Statistical Analysis

We analyzed the frequency of outpatient consultations (including in-person and telemedicine phone consultations), HbA_1c_, estimated glomerular filtration rate (eGFR), and urine albumin-creatinine ratio (UACR), comparing data from April to September 2020 with data from April to September 2019 using Wilcoxon signed rank tests, while changes in dipstick proteinuria were compared using the McNemar test. We used a definition of clinically significant deterioration of HbA_1c_ as an elevation of HbA_1c_ by more than 2% of the median value of HbA_1c_ in 2019 [18]. To evaluate the change in the rate of decline of eGFR, we compared the change of eGFR from 2019 to 2020 (ΔeGFR 2019-2020) with that of the previous year’s change (ΔeGFR 2018-2019) using the Wilcoxon signed rank test. In addition, we conducted a multivariate logistic regression analysis to identify factors related to the changes in glycemic control and eGFR. We also compared the changes in HbA_1c_ and eGFR from 2019 to 2020 among telemedicine users and telemedicine nonusers using difference-in-differences design. Data are presented as mean (SD) or median (IQR). Values of *P*<.05 were defined as statistically significant. Statistical analyses were performed using JMP Pro 16 (SAS Institute Inc).

### Ethical Approval

The study protocol was approved by the Research Ethics Committee of The University of Tokyo (2020267NI). Informed consent by participants were obtained by opt-out approach.

## Results

### Characteristics of Study Participants

We identified 5036 patients who visited the Department of Diabetes and Metabolic Diseases at 1 to 3 months intervals in 2019 and 2020, consisting of 3746 patients with *ICD-10* code E11 (type 2 DM), 1233 with *ICD-10* code E14 (unspecified DM), and 57 with HbA_1c_ levels of 6.5% or higher. After excluding patients who did not fulfil the inclusion criteria, the remaining 3035 patients were included as the study cohort (Figure 1).

The characteristics of the study patients are shown in Table 1. The median age of patients was 70 (IQR 61-77) years, with 37.3% (1131/3035) being female. Dyslipidemia (2406/3035, 79.3%) and hypertension (2079/3035, 68.5%) were the 2 main comorbidities. Telemedicine users were more likely to be female compared with telemedicine nonusers (141/297, 47.5% vs 990/2738, 36.2%; *P*<.001).

**Table 1:** Characteristics of study participants.

Variables		Overall (N=3035)	Telemedicine users (n=297, 9.8)	Telemedicine nonusers (n=2738, 90.2)	*P* value^a^	
Age, median (IQR)		70 (61-77)	71 (61-78)	70 (61-77)	.17	
Female gender, n (%)		1131 (37.3)	141 (47.5)	990 (36.2)	<.001	
**Comorbidities, n (%)**						
	Dyslipidemia	2406 (79.3)	235 (79.1)	2171 (79.3)	.95	
	Hypertension	2079 (68.5)	196 (66)	1883 (68.8)	.33	
	Cardiovascular disease	1734 (57.1)	160 (53.9)	1574 (57.5)	.23	
	Malignancy	1714 (56.5)	171 (57.6)	1543 (56.4)	.69	
	Chronic kidney disease	1235 (40.7)	112 (37.7)	1123 (41)	.27	
	Cognitive Impairment	149 (4.9)	10 (3.4)	139 (5.1)	.17	

^a^Analysis was performed using Wilcoxon rank sum test.

### Phone-Based Telemedicine Consultation Among Outpatients With Diabetes

The total median number of outpatient consultations (both in-person and telemedicine phone consultations) was 4 (IQR 3-4) among telemedicine users, of which the median number of telephone-based telemedicine consultations was 1 (IQR 1-1). The total median number of outpatient consultations was 2 (IQR 2-3) among telemedicine nonusers in 2020, significantly lower than that of telemedicine users (*P*<.001).

### Evaluation of Changes in the Number of Outpatient Consultations, Glycemic Control, and Renal Function Among Outpatients With Diabetes Between the Time Before the Pandemic and the Early Stages of the Pandemic

Table 2 and Table 3 present the changes in frequency of outpatient consultations, HbA_1c_, eGFR, UACR, and dipstick proteinuria among the study patients before and during the COVID-19 pandemic. The overall median number of outpatient consultations decreased significantly from 3 (IQR 2-3) in 2019 to 2 (IQR 2-3) in 2020 (*P*<.001). The frequency of outpatient consultations was between 3-4 for 63.9% (n=1938) of the patients before the pandemic, which is significantly higher than that during the pandemic (n=1354, 45.6%; *P*<.001). The median HbA_1c_ level of 6.95% (IQR 6.47%-7.40%, 95% CI 6.90-6.97) in 2020 (during the pandemic) increased (*P*<.001) compared with the median HbA_1c_ level of 6.90% (IQR 6.47%-7.39%, 95% CI 6.88-6.94) in 2019 (before the pandemic) among the same cohort of patients, but the increase was not clinically significant.

The median eGFR levels declined slightly in 2020 compared to 2019. The decline in median eGFR was significantly greater in the period of 2019-2020 (–0.9, IQR –4.0 to 2.1, 95% CI –1.2 to –0.8 mL/min/1.73 m^2^) compared to 2018-2019 (–0.5, IQR –3.4 to 2.3, 95% CI –0.7 to –0.3 mL/min/1.73 m^2^; *P*=.01). To examine whether the decline in eGFR was transient or sustained in nature, we also analyzed the eGFR of the study cohort for the year 2021. We found that the median eGFR declined further in 2021. The decline in the median eGFR was significantly greater in the period of 2020-2021 (–1.4, IQR –7.4 to –1.4, 95% CI –1.5 to –1.1 mL/min/1.73 m^2^) compared to the period of 2018-2019 (Table S2 and S3 in Multimedia Appendix 1).

The median UACR levels increased significantly (19.0, IQR 11.0-60.5, 95% CI 17.0-21.0 g/gCr; *P*<.001) during the pandemic in 2020. In dipstick proteinuria tests, the number of patients with negative proteinuria decreased from 2076/2737 (75.8 %) patients in 2019 to 1842/2798 (65.8 %) patients in 2020. The number of patients with overt proteinuria increased from 564/2737 (20.6 %) patients in 2019 to 625/2798 (22.3 %) patients in 2020 (*P*<.001).

The adjusted logistic regression analysis indicated that age and HbA_1c_ level during 2019 were positive predictors of worsening glycemic control during COVID-19 in 2020, whereas the number of outpatient consultations attended was identified as a negative predictor of worsening glycemic control (odds ratio 0.89, 95% CI 0.82-0.96; *P*=.004). The logistic regression model also indicated the decline of eGFR (ΔeGFR) and urinary proteinuria during 2019 as positive predictors of worsening glycemic control during the COVID-19 pandemic in 2020 (Table 4).

**Table 2:** Comparison of frequency of outpatient consultations attended (both in person and telephone-based), glycated hemoglobin A_1c_ (HbA_1c_), estimated glomerular filtration rate (eGFR), and urinary albumin creatinine ratio (UACR) among patients with diabetic kidney disease before and during the COVID-19 pandemic.

Variables	Before pandemic (2019)		During pandemic (2020)		Difference (2020-2019), median (95% CI)	*P* value
	Median (IQR)	95% CI	Median (IQR)	95% CI		
Number of outpatient consultations attended (N=3035)	3 (2 to 3)	3.0 to 3.1	2 (2 to 3)	2.6 to 2.7	0 (0 to 0)	<.001^a^
HbA_1c_ (%; N=3035)	6.90 (6.47 to 7.39)	6.88 to 6.94	6.95 (6.47 to 7.40)	6.90 to 6.97	0.033 (0.017 to 0.050)	<.001^a^
eGFR (mL/min/1.73 m^2^; N=3035)	66.1 (54.5 to 77.3)	65.1 to 66.9	64.7 (53.7 to 76.0)	64.0 to 65.4	–0.92 (–1.17 to –0.75)	<.001^a^
ΔeGFR^b^ (mL/min/1.73 m^2^; n=2946)	–0.5 (–3.4 to 2.3)	–0.7 to –0.3	–0.9 (–4.0 to 2.1)	–1.2 to –0.8	–0.33 (–0.67 to 0.00)	.01^a^
UACR (g/gCr; n=858)	19.0 (9.0 to 51.8)	17.0 to 20.7	19.0 (11.0 to 60.5)	17.0 to 21.0	1.0 (0.5 to 2.0)	<.001^a^

^a^Analysis performed using Wilcoxon signed rank tests.

^b^The change of eGFR from 2019 to 2020.

**Table 3:** Percentages of outpatient consultations attendance (both in person and telephone-based) and percentages of patients with negative, trace, and positive proteinuria for dipstick proteinuria tests among patients with diabetic kidney disease before and during the COVID-19 pandemic (N=3035).

Variables				Before pandemic (2019)		During pandemic (2020)		*P* value
**Frequency of outpatient consultations attended, n (%)**								—^a^
	1			72 (2.4)		197 (6.5)		
	2			1025 (33.8)		1484 (48.9)		
	3			1204 (39.7)		879 (29.9)		
	≥4			734 (24.2)		475 (15.7)		
**Dipstick proteinuria tests, n (%)**								<.001^b^
		Negative	2076 (75.8)		1842 (65.8)			
		Trace	97 (3.5)		331 (11.8)			
		Positive (1 to 4)	564 (20.6)		625 (22.3)			

^a^Not applicable.

^b^Analysis performed using the McNemar test.

**Table 4:** Odds ratios (ORs) for deterioration of glycemic control and estimated glomerular filtration rate (eGFR) during the COVID-19 pandemic.

Variables	Glycemic control^a^					eGFR^b^				
	Model 1		Model 2^c^			Model 1			Model 2^c^	
	Crude ORs (95% CI)	*P* value^d^	Adjusted ORs (95% CI)	*P* value^d^	Crude ORs (95% CI)		*P* value^d^	Adjusted ORs (95% CI)		*P* value^d^
Age	1.01 (1.00-1.02)	<.001	1.01 (1.00-1.02)	.002	1.00 (0.99-1.00)		.84	1.00 (0.99-1.01)		.65
HbA_1c_ in 2019	1.11 (1.01-1.22)	.02	1.19 (1.07-1.32)	.001	0.95 (0.87-1.04)		.25	1.01 (0.88-1.15)		.94
ΔeGFR in 2019	0.99 (0.98-1.01)	.47	1.00 (0.98-1.01)	.56	0.64 (0.62-0.66)		<.001	0.64 (0.62-0.67)		<.001
Urinary proteinuria in 2019	1.00 (0.82-1.21)	.97	1.03 (0.84-1.26)	.61	1.06 (0.88-1.28)		.03	1.47 (1.13-1.90)		.007
Number of outpatient consultations attended in 2020	0.90 (0.85-0.97)	.004	0.89 (0.82-0.96)	.003	0.96 (0.90-1.02)		.22	0.96 (0.87-1.06)		.41

^a^Deterioration of glycemic control is defined by elevated hemoglobin A_1c_ (HbA_1c_) level more than 2% from the baseline.

^b^Deterioration of eGFR is defined as larger eGFR decline in 2019-2020 compared to 2018-2019.

^c^Multivariable regression analysis adjusted for the following: age; sex; HbA_1c_ in 2019; ΔeGFR in 2019; urinary proteinuria in 2019; the number of visits in 2020; use of telemedicine; and *International Classification of Diseases, Tenth Revision* codes for chronic kidney disease, cardiovascular disease, cognitive impairment, dyslipidemia, hypertension, and malignancy.

^d^Analysis was performed using multivariable logistic regression.

### Comparison of Glycemic Control and Renal Function During the Early Stages of the Pandemic Between Telemedicine Users and Telemedicine Nonusers

Difference-in-differences analyses showed no significant differences in the change of median HbA_1c_ (0.01%, 95%CI –0.14 to –0.16; *P*=.90) and eGFR (0.6, 95% CI –0.1 to 1.4 mL/min/1.73m^2^; *P*=.10) between telemedicine users (n=297) and telemedicine nonusers (n=2738; Table 5).

**Table 5:** Difference-in-differences analysis to compare glycated hemoglobin A_1c_ (HbA_1c_) and estimated glomerular filtration rate (eGFR) between telemedicine and telemedicine nonusers during the COVID-19 pandemic.

	Telemedicine users (n=297), median (IQR)			Telemedicine nonusers (n=2738), median (IQR)			Difference-in-differences analysis	
	2019	2020	2019		2020	Estimates (95％CI)		*P* value^a^
HbA_1c_ (%)	6.90 (6.30-7.38)	6.90 (6.40-7.42)	6.90 (6.50-7.39)		6.95 (6.50-7.40)	0.01 (–0.14 to 0.16)		.90
eGFR (mL/min/1.73 m^2^)	66.8 (55.7-78.6)	65.7 (54.9-77.4)	66.0 (54.4-77.1)		64.5 (53.6-75.8)	0.6 (–0.1 to 1.4)		<.10

^a^Analysis is done using difference-in-differences technique.

## Discussion

### Principal Findings

In this study, we evaluated changes in the frequency of outpatient consultations, glycemic control, and renal function among a study cohort with type 2 DM before and during the early phase of the COVID-19 pandemic (ie, April to September 2020). We also investigated the utilization rate of telemedicine via phone consultations and compared the glycemic control and renal function among telemedicine users and nonusers during the COVID-19 pandemic. Our study revealed that the frequency of outpatient consultations showed a statistically significant reduction during the COVID-19 pandemic. There was a decline in glycemic control during the first 6 months of the pandemic, although the difference was not clinically significant. Our cohort of patients also experienced acceleration in the sustained decline of renal function during the pandemic over a period of 2 years (2020 and 2021). Next, our study shows that the proportion of the cohort of patients who used telemedicine consultations was only 9.8% (297/3035). Glycemic control and renal function of telemedicine users did not differ much from those who did not attend phone telemedicine consultations during the COVID-19 pandemic.

### Comparison to Prior Work

The decrease in frequency of outpatient consultations from 3 (IQR 2-3) visits before the pandemic to 2 (IQR 2-3) visits during the early phase of the pandemic is considered clinically significant in the context of diabetes care. As patients with well-controlled diabetes typically attend outpatient follow-up visits every 3 months, missing 1 appointment could result in a disruption of continuity of care. Furthermore, it has been reported that missing the last scheduled primary care appointment is associated with an increased risk of hospital admission among patients with diabetes who were recently hospitalized [19,20].

Although there was a decline in glycemic control during the first 6 months of the pandemic, the difference was not clinically significant, as reported previously [21]. Nevertheless, older patients and patients with poor glycemic control should be given extra attention, as we found that advancing age and HbA_1c_ level are associated with worsening glycemic control during COVID-19. Treatment intensification may not have been properly implemented in patients with poor glycemic control due to reduction in outpatient visits. From our study, we also discovered that a reduction in attendance of outpatient consultations was significantly associated with declining glycemic control during COVID-19. Our findings aligned with the evidence that showed the importance of continuity of care in improving glycemic control among patients with diabetes [22].

Our cohort of patients also experienced an acceleration in the sustained decline of renal function during the pandemic over the period of 2 years (2020 and 2021). Our findings also align with those of another study that reported a significant decline in the frequency of physician appointments and a significant increase in the mean creatinine levels among patients with diabetes during the COVID-19 pandemic [23]. Furthermore, since deterioration of renal function during COVID-19 is associated with urinary proteinuria before the pandemic, this group of patients should be closely monitored. Continuity of care from physicians has been shown to reduce renal progression among patients with diabetes [24]; therefore, consistent and regular outpatient care is important for them.

Telemedicine can be implemented by various modalities [25]. In Japan, 72.9% of the telemedical first visits in September 2020 were reported to be via phone calls, and the prevalence of telemedicine use is still quite low, as is the case with this study [26,27]. Our study shows that the proportion of the cohort of patients who used telemedicine consultations was only 9.8% (297/3035), and the number of telephone consultations used was only 1 over the 6-month study period. Due to consistent report of low utilization rate of telemedicine shown in our study as well as other studies, there is a need to increase patients’ awareness of the availability of telemedicine consultation services and educate patients on how to use and benefit from telemedicine consultations. Understanding patients’ barriers to using telemedicine is important, as it has been reported that some older patients were unready for telephone visits because of difficulties in hearing and communication or dementia [28]. Moreover, as telemedicine was not yet widespread in Japan before the COVID-19 pandemic [15], it was possible that health care providers were unfamiliar with the safety and efficacy of implementing telemedicine consultations, and thus, hesitant to provide them.

Our results show that the glycemic control and renal function of patients who attended phone telemedicine consultations did not differ much from those who did not attend phone telemedicine consultations during the COVID-19 pandemic. Our results correspond with those of a study that revealed that the difference in consultation modality (in person or by phone) did not affect glycemic control [29]. Although phone consultation during the pandemic allowed the telemedicine users to have more frequent contact with physicians compared with the telemedicine nonusers, the benefits on the improvement of glycemic control and renal function progression were limited, as shown in our study. This could be due to the infrequent use of phone consultations among the telemedicine users in our study cohort. Another study that implemented weekly phone consultations showed significantly improved overall glycemic control and lipid profile of patients with diabetes [30]. Moreover, an average frequency of once in 6 months for telemedicine consultations via phone alone may not be sufficient for physicians to assess patients’ clinical progression. Compared with phone consultations, video consultations provide some aspects of physical examination and a more personal connection between clinicians and patients [31]. Telemedicine consultation could be coupled with remote monitoring using home self-test kits and self-care assistance via smart phone–based mobile health (mHealth) interventions. In addition to real-time feedback to patients, mHealth facilitates information exchange and interactions between patients and health care providers [32]. Furthermore, the use of smart phone–based mHealth apps is associated with increased patient satisfaction with telemedicine appointments [33]. The combination of different telemedicine modalities may improve quality of care.

### Limitations

There are some limitations in our study. Medical consultations are covered by health insurance for every resident in Japan; our results may not generalize to countries using different health insurance systems. As data were only collected from a single tertiary medical institution located in an urban region in the capital city, generalizability to other Japanese settings should be interpreted with caution due to differences in telemedicine facility and patient management style during COVID-19. The study was limited to the first half year of the pandemic. BMI, blood pressure, and lipid control, critical for the progression of diabetic complications, were not assessed. Decline of renal function is affected by aging, gender, medication therapy, and genetic background [34], and eGFR and HbA_1c_ could be affected by changes in medications. These factors were not considered in our analyses. There is a possibility that COVID-19 infection may cause proteinuria and acute kidney injury [35]; however, we do not have access to information of COVID-19 diagnosis among the study cohort during the study period.

In this study, we only compared the frequency of outpatient consultations before and during the early phase of the pandemic; we did not examine the frequency of other diabetes-related preventive services. We did not examine patient-reported outcomes of diabetes. The small sample size of telemedicine users and the limited number of telemedicine consultations among telemedicine users may affect the results of our findings; therefore, the findings should be interpreted with caution.

Additionally, this study refers to data during the early phase of the COVID-19 pandemic and may not be applicable to the current phase of the pandemic. As the pandemic enters its third year with several countries announcing plans to transition from pandemic control to endemic management of COVID-19 [36], the Japanese government has also loosened COVID-19 restrictions. As of March 2023, the Japanese government has issued an official statement to discontinue the previous deregulations on the use of telemedicine for medical consultations, which will take effect in August 2023 [37]. Nevertheless, this study offers valuable insights on the utility of telemedicine outpatient consultations for patients with diabetes.

### Conclusions

The COVID-19 pandemic led to declines in outpatient consultations among patients with type 2 DM in Japan. Glycemic control of patients was well maintained, but patients experienced rapid declines in renal function during the pandemic. These clinical outcomes did not differ between patients who used telemedicine phone consultations and those who did not. Further studies are needed to explore the effectiveness of different modalities and frequencies of telemedicine consultations for patients with diabetes.

## Appendix

Multimedia Appendix 1 Supplementary material.

## Abbreviations

DM: diabetes mellitus
eGFR: estimated glomerular filtration rate
HBA_1c_: hemoglobin A_1c_
ICD-10: International Classification of Diseases, Tenth Revision
mHealth: mobile health
UACR: urine albumin creatinine ratio
