# Correction: Glycemic Control, Renal Progression, and Use of Telemedicine Phone Consultations Among Japanese Patients With Type 2 Diabetes Mellitus During the COVID-19 Pandemic: Retrospective Cohort Study

**DOI:** 10.2196/72076

**Published:** 2025-03-06

**Authors:** Akiko Sankoda, Yugo Nagae, Kayo Waki, Wei Thing Sze, Koji Oba, Makiko Mieno, Masaomi Nangaku, Toshimasa Yamauchi, Kazuhiko Ohe

**Affiliations:** 1 Department of Planning, Information and Management The University of Tokyo Hospital Tokyo Japan; 2 Department of Diabetes and Metabolic Diseases, Graduate School of Medicine The University of Tokyo Tokyo Japan; 3 Department of Biomedical Informatics, Graduate School of Medicine The University of Tokyo Tokyo Japan; 4 Department of Biostatistics, School of Public Health, Graduate School of Medicine The University of Tokyo Tokyo Japan; 5 Department of Medical Informatics, Center for Information Jichi Medical University Shimotsuke Japan; 6 Division of Nephrology and Endocrinology, Graduate School of Medicine The University of Tokyo Tokyo Japan

In “Glycemic Control, Renal Progression, and Use of Telemedicine Phone Consultations Among Japanese Patients With Type 2 Diabetes Mellitus During the COVID-19 Pandemic: Retrospective Cohort Study” (JMIR Diabetes 2023;8:e42607) the authors made one addition.

The equal contribution footnote (marked by *) was added for the authors Akiko Sankoda and Yugo Nagae. The final authorship list appears as follows:

Akiko Sankoda^1^*, Yugo Nagae^1*^, Kayo Waki^1,2,3^, Wei Thing Sze^2^, Koji Oba^4^, Makiko Mieno^5^, Masaomi Nangaku^6^, Toshimasa Yamauchi^3^, Kazuhiko Ohe^1,2^

*these authors contributed equally

The correction will appear in the online version of the paper on the JMIR Publications website on March 6, 2025, together with the publication of this correction notice. Because this was made after submission to PubMed, PubMed Central, and other full-text repositories, the corrected article has also been resubmitted to those repositories.

