# Supplementary material for: Glycemic Control, Renal Progression, and Use of Telemedicine Phone Consultations Among Japanese Patients With Type 2 Diabetes Mellitus During the COVID-19 Pandemic: Retrospective Cohort Study
Source: JMIR Diabetes. 2023 Nov 21;8:e42607. doi: 10.2196/42607 (PMC10698649; doi:10.2196/42607)
Supplement: Multimedia Appendix 1 [file diabetes_v8i1e42607_app1.docx]

Table S1: Medical Complications according to ICD-10 code.

| Disorders | ICD-10 code |
| --- | --- |
| Dyslipidemia | E106,E116,E146,E755,E780,E782,E784,E785,E786,E789 |
| Hypertension | D350,E790,F453,G448,H208,H350,I10,I110,I119,I120,I129,I139,I150,I151,I152,I159,I270,I272,I619,I674 |
| Cardiovascular disease (CVD) | D688,E059,G450,G458,G459,G463,G467,I099,I10,I110,I119,I139,I200,I201,I208,I209,I210,I211,I212,I213,I214,I219,I220,I221,I228,I229,I230,I231,I232,I233,I234,I235,I236,I238,I241,I249,I251,I252,I253,I255,I256,I259,I438,I500,I501,I509,I519,I601,I604,I606,I608,I609,I610,I611,I613,I614,I615,I616,I619,I630,I631,I632,I633,I634,I635,I636,I638,I639,I64,I651,I653,I660,I661,I662,I663,I669,I670,I671,I672,I674,I676,I677,I678,I679,I691,I693,I694,I709,I725,I731,I738,I739,M303 |
| Chronic Kidney Disease (CKD) | B169,B171,B238,D638,D690,D868,E102,E112,E132,E142,E274,E790,E850,E888,H328,I120,I129,I139,I701,I823,K767,M1009,M321,M348,N002,N003,N004,N007,N009,N012,N014,N017,N019,N028,N029,N030,N032,N033,N034,N037,N039,N042,N044,N051,N052,N053,N054,N055,N056,N057,N058,N059,N078,N079,N080,N082,N083,N085,N088,N110,N111,N118,N119,N12,N140,N141,N142,N143,N144,N181,N182,N183,N184,N185,N189,N19,N250,N251,N258,N26,N270,N271,N279,N280,N281,N288,N289,N298,N391,Q249,Q271,Q600,Q605,Q610,Q611,Q612,Q613,Q614,Q615,Q618,R944,T861,Y459,Y575,Z905,Z940,Z992 |
| Cognitive Impairment | A504,A521,A810,B220,E52,E538,E756,E830,F000,F001,F002,F009,F010,F011,F012,F019,F020,F022,F023,F024,F028,F03,F051,F067,F107,F843,G10,G20,G238,G300,G301,G308,G309,G310,G318,G328 |
| Malignancy | B210,C000,C001,C002,C003,C004,C006,C008,C009,C01,C020,C021,C022,C029,C030,C031,C039,C040,C041,C049,C050,C051,C052,C059,C060,C061,C062,C069,C07,C080,C081,C089,C090,C091,C099,C100,C101,C102,C103,C104,C109,C110,C111,C112,C113,C119,C12,C130,C131,C132,C139,C140,C150,C151,C152,C153,C154,C155,C158,C159,C160,C161,C162,C163,C164,C165,C166,C169,C170,C171,C172,C179,C180,C181,C182,C183,C184,C185,C186,C187,C189,C19,C20,C210,C211,C220,C221,C223,C224,C227,C229,C23,C240,C241,C248,C249,C250,C251,C252,C253,C254,C257,C258,C259,C261,C300,C301,C310,C311,C312,C313,C319,C320,C321,C322,C329,C33,C340,C341,C342,C343,C348,C349,C37,C380,C381,C382,C383,C384,C400,C410,C411,C440,C441,C442,C443,C444,C445,C446,C447,C449,C450,C451,C452,C459,C482,C493,C500,C501,C502,C503,C504,C505,C506,C508,C509,C510,C511,C512,C519,C52,C530,C531,C538,C539,C540,C541,C543,C549,C55,C56,C570,C579,C58,C600,C601,C602,C609,C61,C629,C630,C632,C637,C639,C64,C65,C66,C670,C671,C672,C673,C674,C675,C676,C677,C679,C680,C695,C73,C740,C749,C750,C760,C761,C780,C782,C783,C784,C786,C787,C791,C792,C793,C794,C795,C796,C798,C799,C800,C809,C859,D000,D001,D002,D010,D011,D012,D013,D014,D019,D020,D021,D022,D023,D040,D041,D043,D044,D045,D046,D047,D049,D050,D051,D059,D060,D061,D069,D070,D071,D072,D073,D074,D075,D090,D092,D099,D137,D372,D377,D630,E890,F452,G130,G992,H350,J178,K210,K227,K768,K918,L270,L984,Q859,R11,R522,R798,T451,T825,T889,Z080,Z512,Z988,Z992 |

Table S2: Trend of eGFR value from year 2018-2021

|  | 2018 | 2019 | 2020 | 2021 |
| --- | --- | --- | --- | --- |
| Median | 66.1 | 66.1 | 64.7 | 63.5 |
| IQR | 55.3-77.8 | 54.5-77.3 | 53.7-76.0 | 52.3-75.1 |
| 95% CI | 65.5-66.8 | 65.1-66.9 | 64.0-65.4 | 62.2-63.7 |

Table S3: Comparison of between-year eGFR decline from 2018-2021

|  | 2018-2019 | 2019-2020 | 2020-2021 |
| --- | --- | --- | --- |
| Median | -0.5 | -0.9 | -1.4 |
| IQR | -3.4- 2.3 | -4.0- 2.1 | -7.4- -1.4 |
| 95%CI | -0.7- -0.3 | -1.2- -0.8 | -1.5- -1.2 |
| P value | reference | 0.118 | <.001 |
